# Different feeding strategies in Antarctic scavenging amphipods and their implications for colonisation success in times of retreating glaciers

**DOI:** 10.1186/s12983-017-0248-3

**Published:** 2017-12-27

**Authors:** Meike Anna Seefeldt, Gabriela Laura Campana, Dolores Deregibus, María Liliana Quartino, Doris Abele, Ralph Tollrian, Christoph Held

**Affiliations:** 1 Department of Animal Ecology, Evolution and Biodiversity, Ruhr-University Bochum, Bochum, Germany; 2 Alfred-Wegener-Institut Helmholtz- Zentrum für Polar und Meeresforschung, Bremerhaven, Germany; 3 Departamento de Biología Costera, Instituto Antártico Argentino, Buenos Aires, Argentina; 4 Departamento de Ciencias Básicas Universidad Nacional de Luján, Luján, Argentina; 5 Museo Argentino de Ciencias Naturales “B. Rivadavia”, Buenos Aires, Argentina

**Keywords:** Southern Ocean, King George Island/ Isla 25 de Mayo, Potter Cove, Succession, Carrion-feeding, Food web, *Notothenia rossii*, *Notothenia coriiceps*, *Palmaria decipiens*, *Desmarestia menziesii*

## Abstract

**Background:**

Scavenger guilds are composed of a variety of species, co-existing in the same habitat and sharing the same niche in the food web. Niche partitioning among them can manifest in different feeding strategies, e.g. during carcass feeding. In the bentho-pelagic realm of the Southern Ocean, scavenging amphipods (Lysianassoidea) are ubiquitous and occupy a central role in decomposition processes. Here we address the question whether scavenging lysianassoid amphipods employ different feeding strategies during carcass feeding, and whether synergistic feeding activities may influence carcass decomposition. To this end, we compared the relatively large species *Waldeckia obesa* with the small species *Cheirimedon femoratus, Hippomedon kergueleni,* and *Orchomenella rotundifrons* during fish carcass feeding (*Notothenia* spp.). The experimental approach combined ex situ feeding experiments, behavioural observations, and scanning electron microscopic analyses of mandibles. Furthermore, we aimed to detect ecological drivers for distribution patterns of scavenging amphipods in the Antarctic coastal ecosystems of Potter Cove. In Potter Cove, the climate-driven rapid retreat of the Fourcade Glacier is causing various environmental changes including the provision of new marine habitats to colonise. While in the newly ice-free areas fish are rare, macroalgae have already colonised hard substrates. Assuming that a temporal dietary switch may increase the colonisation success of the most abundant lysianassoids *C. femoratus* and *H. kergueleni*, we aimed to determine their consumption rates (g food x g amphipods^−1^ x day^−1^) and preferences of macroalgae and fish.

**Results:**

We detected two functional groups with different feeding strategies among scavenging amphipods during carcass feeding: carcass ‘opener’ and ‘squeezer’. Synergistic effects between these groups were not statistically verified under the conditions tested. *C. femoratus* switched its diet when fish was not available by consuming macroalgae (about 0.2 day^−1^) but preferred fish by feeding up to 80% of its own mass daily. Contrary, *H. kergueleni* rejected macroalgae entirely and consumed fish with a maximal rate of 0.8 day^−1^.

**Conclusion:**

This study reveals functional groups in scavenging shallow-water amphipods and provides new information on coastal intraguild niche partitioning. We conclude that the dietary flexibility of *C. femoratus* is a potential ecological driver and central to its success in the colonisation of newly available ice-free Antarctic coastal habitats.

**Electronic supplementary material:**

The online version of this article (10.1186/s12983-017-0248-3) contains supplementary material, which is available to authorized users.

## Background

In order to understand the factors responsible for the evolutionary basis of biodiversity patterns and ecosystem functioning it is important to understand species interactions and adaptations. Different feeding types such as carnivory, necrophagy, herbivory and omnivory have evolved as adaptive traits to the ecosystems and the available niches therein.

Scavenger guilds, feeding on dead animal and/or plant biomass, are present in all ecosystems and are composed of a diversity of co-existing species in the same habitat. The predominant functional role of scavengers in an ecosystem is to recycle biomass for energy transfer through the food web. It is worth noticing that careful attention should be paid while using the terms ‘scavenger’, ‘carrion feeder’ (≙ carcasivore), ‘litter feeder’ (≙ lectivore) and ‘detritus feeder’ (≙ decomposer) [1]. Carrion-feeders are specialized on animal carcasses while lectivores are feeding on dead plant material such as leaf-litter and dead wood [1–3]. Scavengers can either exclusively feed on dead material (obligate) or, alternatively, employ other feeding strategies such as predation (facultative). In contrast, decomposers are feeding on the scavengers´ leftovers, i.e. detritus, and hence are not considered ‘scavengers’ [1]. A consistent use and differentiation of these terms is of particular significance for analyses such as food web modelling [1].

Niche partitioning between competing carrion feeders manifests in morphological and behavioural adaptations with a well-known terrestrial example being the avian scavengers. The succession, functional role, and the complementary influence of avian scavengers, particularly vultures, during carcass feeding have been the subject of several studies and conservation management strategies [4–8].

In marine environments, the decomposition of carcasses shows a successional pattern, mainly initiated by quick and highly motile species. In the first successional stage, crustacean amphipods from the superfamily Lysianassoidea play a dominant role throughout the marine realm [9–11]. Especially in the Southern Ocean lysianassoid amphipods are amongst the most ubiquitous taxa of the Antarctic bentho-pelagic realm [12, 13]. Due to their predominantly scavenging feeding mode and high abundance, they occupy an essential role in Antarctic food webs [14]. For deep-sea amphipods, functional groups of lysianassoids have already been characterised based primarily on mandible morphology [15, 16]. The significance of this mouthpart character in feeding mode analyses has been established for decades [15, 17, 18]. However, knowledge of the functional roles of members of the Antarctic scavenging intraguild of lysianassoid amphipod during carrion feeding is scant. It is important to identify functional groups and understand the functional roles among scavenging amphipods during carcass decomposition in order to get a better understanding of community structure, ecosystem functioning, and development of biodiversity patterns. This is particularly important for Antarctic coastal environments that are highly affected by climate driven environmental change.

The marine (eco)system of Potter Cove (South Shetland Islands, Antarctica, PC) is undergoing climate-driven environmental change which is mainly caused by the remarkably fast retreat (approx. 40 m per year) of the Fourcade Glacier- a tidewater glacier which has now retreated landward (pers. observation). Colonization of newly available ice-free hard and soft substrates in the inner cove by advancing macroalgae [19] and zoobenthos [20–22] have already been detected.

In order to understand the functioning of the scavenging amphipod intraguild of PC, we studied the functional roles scavenging amphipods attain during carcass decomposition. More specifically, we investigated whether the relatively large species *Waldeckia obesa* s.str. (Chevreux 1905) [23, 24] and the small scavenging lysianassoid species *Cheirimedon femoratus* (Pfeffer 1888), *Hippomedon kergueleni* (Miers 1875) and *Orchomenella rotundifrons* K.H. Barnard 1932 can be classified into different functional groups, by conducting behavioural observations, estimations on consumption rates and scanning electron microscopic (SEM) mandible analyses. We hypothesised a higher efficiency of carcass decomposition when large and small species from potentially different functional groups were feeding simultaneously. As a previous study revealed a ubiquitous distribution and high relative abundances of the lysianassoid species *C. femoratus* in outer and inner PC [24], we aimed to find possible ecological drives for this pattern. We expect lysianassoid species that can switch to a leaf-litter feeding strategy (lectivory) to be more successful in colonising newly ice-free areas. To this end, we estimated species-specific consumption rates and preferences towards different food items (macroalgae: *Palmaria decipiens* (Reinsch) R.W. Ricker 1987, fish: *Notothenia rossii* Richardson 1844). Similarly, consumption rates were estimated of another abundant scavenger species (*H. kergueleni*), and with a narrower distribution, on macroalgae (*P. decipiens*, *Desmarestia menziesii* J Agardh 1848) and fish (*Notothenia coriiceps* Richardson 1844)). All this aimed at understanding the present and future biodiversity patterns and successional processes in rapidly changing Antarctic coastal ecosystems.

## Methods

### Study area and sampling

The study was conducted at the *Dallmann laboratory* at *Carlini* station, situated at PC (62°14’S 58°42’W), an approximately. 4 km long and 2.5 km wide tributary inlet of Maxwell Bay on King George Island/Isla 25 de Mayo (South Shetland Islands). PC is divided into inner and outer cove by a glacial moraine and further moraines divide the inner cove into sections [25]. For further information on glaciology [26], hydrography [27, 28] and community structure [19, 22, 29] of PC, a compilation of recent publications on diverse research topics of PC is available on the webpage of the international and interdisciplinary research network IMCONet [30].

**Fig. 1:**
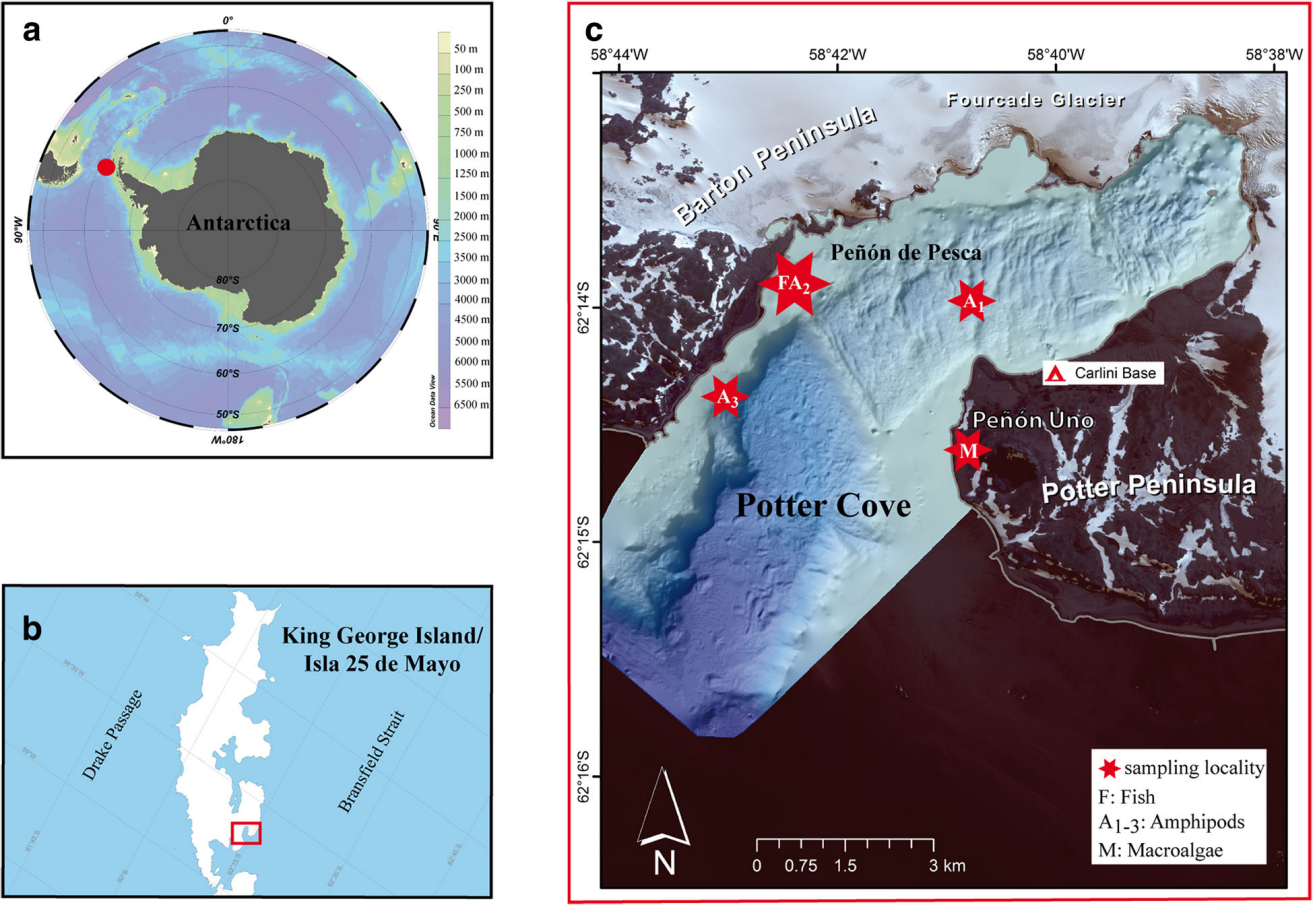
Locality and sampling overview. **a** Location of King George Island/Isla 25 de Mayo **b** Location of Potter Cove on King George Island **c** Sampling stations of Potter Cove. Map information: 1A: Ocean Data View; 1B: SCAR Antarctic Digital Database; 1C: DIGITALGLOBE 2014, WorldView-2 scene 103001001F612100; image courtesy of/copyright DigitalGlobe, Longmont, Co, USA. All rights reserved. Catalogue ID: 103001001F612100, Acq Date: 2013/03/07, Sensor:WV02, Band Info: Pan_MS1_MS2, Resolution0.5 × 0.5 m; ESRI, DigitalGlobe,GeoEye, Earthstar Geographics, CNES/Airbus DS, USDA, USGS, AEX, Getmapping, Aerogrid, IGN, IGP, swisstopo, and the GIS User Community

Lysianassoid amphipods were either collected using baited traps or as bycatch in trammel nets while feeding on dead or moribund specimens of the blackfin icefish *Chaenocephalus aceratus* (Lönnberg 1906). In the first case, dead Antarctic notothenioid fish (*N. rossii*; *N. coriiceps*) were offered as bait. The traps were deployed in the austral summer 2015 and 2016 between 30 m and 35 m depth for 24 h to 48 h (Table 1; Fig. 1c). Amphipods were transferred to 60 l and 80 l aquaria connected to a running seawater flow-through system directly connected to PC shallow waters. Amphipods were fed with fish muscle tissue *ad libitum*. Macroalgae (*Desmarestia* sp.) and pieces of plankton mesh (1000 μm) of random sizes were offered as substrate. Fish (*N. coriiceps* and *N. rossii*) of similar size (*n* = 16; mean: 22.5 cm) were collected using a trammel net (15 m long, 1.5 m deep, 2.5 cm inner mesh, 12 cm outer mesh) in the fishing grounds (Peñon de Pesca) in the outer cove (Table 1). Dead fish bodies were frozen (−18 °C) and completely defrosted in cold filtered seawater 24 h prior to experimentation. The fishes were collected and provided by scientists of the Ichthyology Division of the Argentinean Antarctic Institute.

**Table 1:** Information on the sampling stations to collect lysianassoid amphipods, macroalgae and fish for this study

Station ID	Locality	Coordinates	Depth	Date
A_1_	King George Island, Potter Cove	62°13′57.5S 58°40′52.0 W	30 m	05.01.2015
A_2_	King George Island, Potter Cove, Peñon de Pesca	62° 13S 58° 42 W	30–35 m	Nov 2014; Jan 2016
A_3_	King George Island, Potter Cove	62° 14′23.4S 58° 41′52.3 W	30 m	02.01.2015
F	King George Island, Potter Cove, Peñon de Pesca	62° 13S 58° 42 W	10 m (avg.)	Nov/Dec 2014; Jan 2016
M	King George Island, Potter Peninsula, Peñon Uno	62°14S 58°41 W	shore line	07.01.2015; Jan 2016

Macroalgae (*P. decipiens* and *D. menziesii*) were collected during low tide in the intertidal flotsam Potter Peninsula (Fig. 1c) and maintained in tanks with running seawater in Dallmann laboratory prior to use.

#### Feeding interaction of large and small scavenging lysianassoid species during fish carcass feeding.

**Fig. 2:**
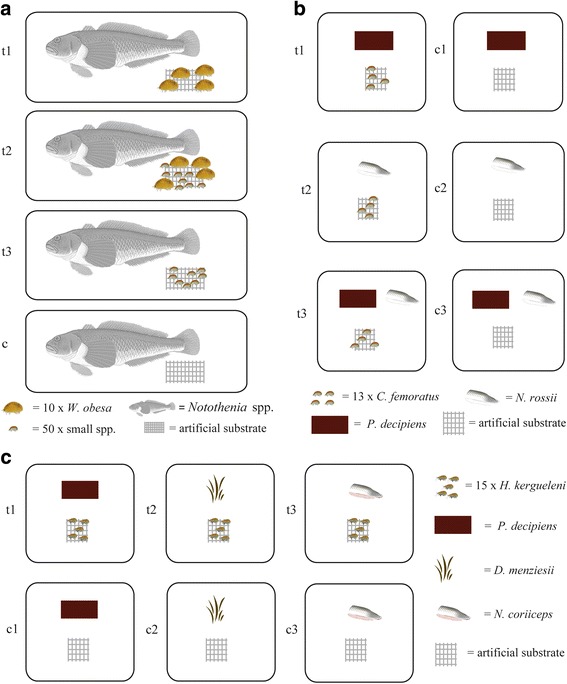
Experimental setup of feeding trials. **a** Consumption rates of scavenging amphipod species on fish carcass. t1:Treatment 1, *n* = 4: *W. obesa*); t2:Treatment 2, n = 4: *C. femoratus*, *H. kergueleni*, *O. rotundifrons*, large and small (t3:Treatment 3, n = 4: *W. obesa, C. femoratus*, *H. kergueleni*, *O. rotundifrons*) and no (control, n = 4) **b** Consumption rates and preference of *C. femoratus* on different food items (t1:Treatment 1, *n* = 12, macroalga: *P. decipiens; Treatment 2*, n = 12, fish: *N. rossii*; Treatment 3, n = 12, choice between *P. decipiens* and *N. rossii;* c1–4 controls) **c** Consumption rates of *H. kergueleni* on different food items (Treatment 1, n = 12, macroalga *P. decipiens*; Treatment 2, n = 12, macroalga *D. menziesii*; Treatment 3, n = 12, fish *N. coriiceps*; C1–3 controls)

To investigate the functional role of large and small amphipod species during carrion feeding, feeding bioassays including four experimental treatments were performed (Fig. 2a). All treatments were conducted in separate 18 l aquaria, filled with triple filtered seawater (10 μm, 5 μm, 0.1 μm). The experimental aquaria were permanently supplied with oxygen by air outlets. Water temperatures among the aquaria ranged between −0.5 °C and +1 °C. In the first treatment, 40 similar-sized randomly selected adult specimens of the assumingly obligate carrion feeder and relative large (2 cm to 3 cm) amphipod *W. obesa* s.str. Were transferred into the experimental aquarium. A similar biomass of amphipods, representing a total of 400 similar-sized adult specimens of the smaller (0.5 cm to 1 cm) amphipod species *C. femoratus, H. kergueleni* and *O. rotundifrons*, were placed in a second aquarium. To test a potential synergistic effect between small and large scavenging amphipod species, 40 adult specimens of *W. obesa* and 400 adult specimens of small amphipod species (*C. femoratus, H. kergueleni, O. rotundifrons*) were added to a third aquarium. A control treatment without amphipods was set up to estimate changes of carcass mass and decomposition due to autogenic effects. For acclimatization and starvation, all amphipods were placed in experimental aquaria 24 h prior experimentation. In order to offer the animals an alternative settling substrate, a piece of plankton mesh (12 × 10 cm, mesh size 1000 μm) was placed in every experimental aquarium.

The fish carcasses were paper dried, weighed with a Sartorius LA 5200D scale to the nearest 0.1 g, and photographed using a Panasonic Lumix DMC-TZ36 digital camera.

The experiment started when the carcasses were transferred into the aquaria and lasted 96 h. Observations of positions, arrangement and movements of amphipods in each aquarium were undertaken and documented several times a day. Additionally, every 12 h amphipod arrangements were visually recorded using the digital camera Panasonic Lumix DMC-TZ36 and the action camcorder Rollei Bullet 5S 1080p. Pictures were analysed using ImageJ software [31] and behavioural observations summarized (Additional file 1). Due to technical problems, i.e. memory failure, no pictures could be analysed of the moment after 24 h.

The aquaria were cleaned (removal of faecal pellets, protein foam) and a partial water change was carried out every 24 h.

After 96 h carcasses were removed from the aquaria and adhering, burrowing amphipod specimens were carefully removed with spring steel forceps. Subsequently, the carcasses were paper dried, pictured and weighed following the same procedure as prior to the experiment. Amphipod biomass (wet mass) was determined to the nearest 0.001 g.

For each treatment, four replicates were included. Both, due to logistical restrictions and sampling limitations (weather conditions) it was not possible to run all replicates simultaneously, so that they were carried out consecutively [32, 33].

Consumption rates (CR) were calculated as g of food consumed per g of amphipod during 24 h (g food x g amphipods^−1^ x day^−1^) following a previous publication [33] using the formula:

$$ CR=\frac{W_{initial}\left({C}_{final}/{C}_{initial}\right)-{W}_{final}}{W_{total}} $$

where W_initial_ and W_final_ are the initial and final blotted wet masses of food in the treatment; C_initial_ and C_final_ are the initial and final masses of the controls; and W_total_ is total biomass of all individuals combined in each treatment. The term C_final_/C_initial_ is a correction factor that serves to consider how much the mass of a fish carcass in the water changes over time from the initial value.

Statistical analysis was performed by testing the data for normality and analysed using a nonparametric Kruskal-Wallis-ANOVA. Differences between the individual groups were analysed by multiple comparisons of mean ranks [34].

#### Consumption rates and feeding preference of *C. femoratus*

In order to assess the consumption rates and feeding preference of *C. femoratus*, no-choice (either fish or macroalgae were offered) and choice (fish and macroalgae were offered) feeding bioassays were performed (Fig. 2b). Thirteen similar-sized adult specimens of *C. femoratus* were randomly assigned to each replicate. The total biomass (wet mass, paper blotted) of amphipods in each treatment was measured to the nearest 0.001 g. For acclimatization and starvation, amphipods were transferred to the experimental containers (275 ml) 24 h prior the experiment. Containers were filled with triple filtered (10 μm, 5 μm, 0.1 μm) sea water and continuously oxygenated with air outlets. A piece of plankton mesh (2 cm × 3 cm, 1000 μm) was added to each container as settling substrate. Following starvation time, water was partly exchanged with fresh filtered seawater and faecal pellets were removed. The water temperature ranged between 0.8 °C and 1.3 °C.

Food items were cut into pieces of similar shapes, paper blotted to remove surplus water, and weighed to the nearest 0.001 g. The mass of food items ranged from 0.138 g to 0.253 g wet mass with a mean of 0.198 g (*n* = 48) for *P. decipiens* and from 1.801 g to 2.875 g wet mass with a mean of 2.202 g (*n* = 48) for *N. rossii*. Each food item (fish, algae, or a piece of both, fish and algae) was placed in individual experimental container with and without amphipods (control) for 24 h. After the experiment, food items were removed from the containers, blotted dry and wet weighed again to the nearest of 0.001 g. The calculation of consumption rates followed the equation given above, according to [33].

The experiments included four replicates of each treatment and were repeated three times so that a total of 12 replicates for each treatment could be conducted. Due to logistical restrictions, it was not possible to run all replicates simultaneously [32, 33].

The data showed no normal distribution. Thus statistical analyses of the differences between two food items and food combinations were carried out using the Mann-Whitney U-test [34].

#### Consumption rates of *H. kergueleni*

To determine consumption rates for different food items (fish: *N. coriiceps* and macroalgae: *P. decipiens*, *D. menziesii*) by the scavenging amphipod *H. kergueleni*, similar feeding assays were conducted (Fig. 2c). Due to logistic constraints (e.g. time, weather, and laboratory capacity), the set up was designed only in terms of no-choice experiments. The volume of containers differed (1200 ml) and number of specimens (15) compared to the design described for *C. femoratus* above. The mass of food items ranged from 0.364 g to 0.536 g wet mass with a mean of 0.447 g (*n* = 24) for *P. decipiens*, from 0.333 g to 0.517 g wet mass with a mean of 0.418 g (*n* = 24) for *D. menziesii*, and from 2.222 g to 3.168 g wet mass with a mean of 2.633 g (*n* = 24) for *N. coriiceps*. However, other conditions (temperatures, procedures, number of replicates per treatment, calculation of consumption) were constant, and statistics were applied as described for the feeding assays of *C. femoratus*.

#### Scanning electron microscopy of mandibles

Selected specimens preserved in 96% ethanol, of *W. obesa*, *C. femoratus, H. kergueleni* and *O. rotundifrons* were dissected using the stereomicroscope Olympus SZH DF PLAPO at Ruhr-University Bochum. To remove remains of food from the dissected mouthparts, a soft paintbrush was used followed by a standardised cleaning procedure [35]. The protocol requires the incubation of mouthparts at room temperature for ten minutes in decreasing ethanol concentrations of 85, 70, 50 and 35% respectively. Subsequently, a COREGA® tab was dissolved in 200 ml demineralised water and samples were transferred into 1 mL COREGA® tab –water solution followed by an ultrasonic bath for 20 s. The samples were washed three times using demineralised water, again sonicated (10 s) and finally washed. Subsequently, samples were incubated in a series of increasing ethanol concentrations (35, 50, 75, 80%) for ten minutes and transferred into their final preservation medium (96% ethanol).

The required gentle dehydration of the delicate samples was achieved using hexamethyldisilazane (HMDS) and acetone. Firstly, samples were incubated in acetone for 20 min and subsequently transferred into a HMDS/acetone solution (50:50) until the HMDS had evaporated. The residual acetone was removed and the samples were again transferred into HMDS until its complete evaporation.

Samples were mounted on SEM stubs and coated with gold (150 s) using the sputter coater SCD 050 (Balzer). Scanning electron microscopic images were produced using the scanning electron microscope DSM 950 (Zeiss).

## Results

### Consumption rates and behaviour of large and small scavenging lysianassoid species during fish carcass feeding

**Fig. 3:**
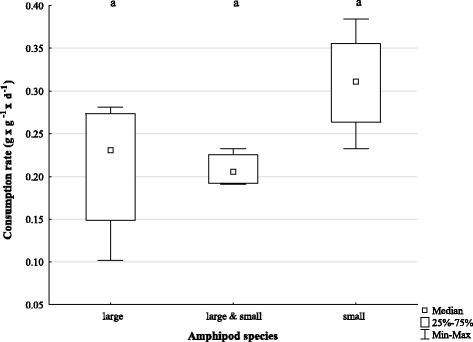
Consumption rates of scavenging amphipods of different species compositions on fish carcass. Amphipod species composition; large: *W. obesa*; large & small: *W. obesa*, *C. femoratus*, *H. kergueleni* and *O. rotundifrons*; small: *C. femoratus*, *H. kergueleni* and *O. rotundifrons*

In general, significant differences were detected among the consumption rates of small (*C. femoratus*, *H. kergueleni*, *O. rotundifrons*), large (*W. obesa*), and the combination of small and large lysianassoid amphipod species on fish carcasses (KW-H (2, *N* = 12) =6.000000 *p* = 0.0498, Fig. 2). However, the multiple comparisons of mean ranks did not confirm significant differences at *p* < 0.05 between the analysed groups. Nonetheless, the data showed a trend between the groups, in which the consumption rate of small amphipods alone reached higher maxima with about 0.40 day^−1^ compared to both the maximal consumption rates of the larger *W. obesa* when feeding alone (0.28 day^−1^), or the combination of small and large amphipod species (0.23 day^−1^; Fig. 3). Consequently, a synergistic effect between large and small amphipod on the speed of carcass decomposition was not statistically confirmed under the conditions tested.

Differences in behaviour of small and large amphipods were observable in all treatments. Immediately after placing the carcass into the aquaria, the small amphipod species started to swim very fast in the tank and partly immediately settled on the carcass. In contrast, the large *W. obesa* responded much slower and calmer to the addition of food by not swimming towards the carcass instantly (Additional file 1).

The positioning of small amphipods on the carcass during the experiment varied slightly among the experimental set-ups regarding their aggregation behaviour. To a lesser extent, visible aggregations of small amphipods occured when *W. obesa* was absent, as most of them were located inside of orifices such as anus, eyes and gill. When *W. obesa* was present, aggregation of small amphipods were observed particularly on caudal and dorsal fins (Additional file 1). However, small amphipods did also feed on the fins when *W. obesa* was present and were also located in the orifices when *W. obesa* was absent only less aggregated. Contrary, no clear aggregation behaviourwas observed for *W. obesa*, which distributed almost evenly on the carcass.

**Fig. 4:**
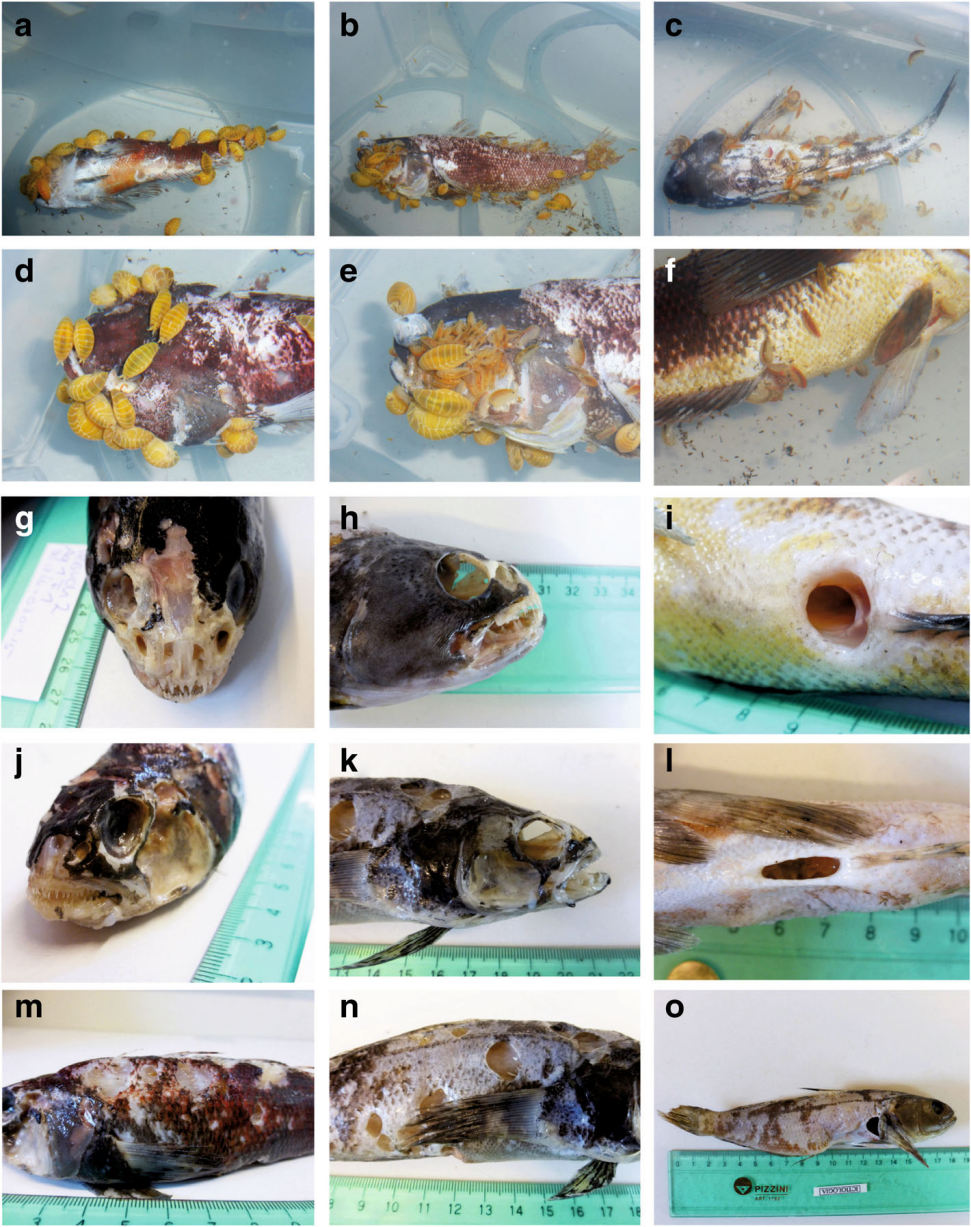
Amphipod agglomeration during carcass feeding and feeding marks. **a**-**f** Agglomeration of scavenging amphipod species after 48 h of feeding on *Notothenia* spp. carcasses **a** + **d**) large species (*W. obesa*) only **b** + **e**) large and small species (*C. femoratus*, *H. kergueleni*, *O. rotundifrons*) **c** + **f**) small species only **G**-**O**) Amphipod feeding marks on *Nothothenia* spp. carcasses after 96 h **g**, **j**, **m**) *large species* **h**, **k**, **n**) large and small species **i**, **l**, **o**) small species only

The feeding marks on fish carcasses after 96 h showed different patterns according to absence or presence of *W. obesa.*, Carcasses exposed to simultaneous feeding of large and small scavengers showed feeding marks in the head region but also along the body and fins (Fig. 4). Head parts of the fish were skeletonised, including mouth and buccal area. Orific such as the eyeswere hollowed out and filled with small amphipods during the experiment. Aggregations of small amphipods were further noticed at the fins, to feed on the thin skin tissue between the rays. In contrast to these observations, specimens of *W. obesa* first opened the thicker body skin and subsequently fed on the muscles, and partly dug into the flesh producing deep feeding marks (Fig. 4). Additionally, individuals of *W. obesa* also fed on the head area but no clear aggregation compared to the rest of the carcass was observed. The distribution of *W. obesa* on the carcass was uniform throughout the experiments. Fish carcasses, which were not co-exposed to small amphipods, showed deep feeding marks throughout the carcass and skeletonised head parts, particularly maxillae. In absence of *W. obesa,* no deep feeding marks on the body and skin of the carcass were observed but small amphipod species fed on carcasses from the inside, entering through the body cavities anus, eyes and gill-opening (Fig. 4). The fish carcass showed sunken abdomen caused by consumption of intestine and muscles, leaving the skin untouched.

Neither cannibalism nor predation among amphipod species was observed during any of these experiments.

### Feeding preference and consumption rates of *C. femoratus*

**Fig. 5:**
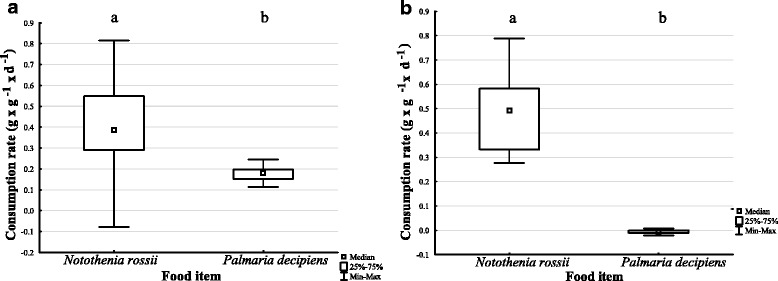
Consumption rate of *C. femoratus* on fish (*N. rossii*) and macroalgae (*P. decipiens*) **a**) no-choice **b**) choice

The analysis of consumption rates and food preference of the most abundant scavenging amphipod species in PC *C. femoratus* [24] indicated a clear preference for fish (MWU: *n* = 12; *p* = 0.006099). While *C. femoratus* consumed up to 80% of its own mass daily (0.8 day^−1^) of fish muscle *(N. rossii)* in the food choice trial, the macroalgae *P. decipiens* remained practically untouched (Fig. 5b, MWU: n = 12; *p* = 0.000037). Contrary, when exclusively left to feed on the macroalgae in the non-choice feeding trials, *C. femoratus* satiated with the vegetarian diet (Fig. 5a) by consuming about 20% of its own mass daily (0.2 day^−1^). Nonetheless, the consumption rate on fish muscle was significantly higher (MWU: n = 12; *p* = 0.006099) than on the macroalgae and with a maximum rate of 0.8 day^−1^ similar compared to the choice trial.

### Consumption rates of *H. kergueleni* on different food items

**Fig. 6:**
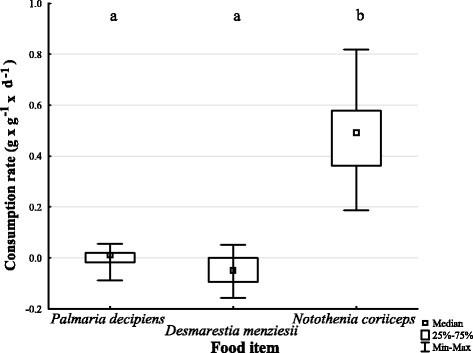
Consumption rate of *H. kergueleni* on macroalgae (*P. decipiens*, *D. menziesii*) and fish (*N. coriiceps*)

The consumption rate of *H. kergueleni* varied significantly when offered different two macroalgae species (*P. decipiens, D. menziesii*) or fish (*N. coriiceps*) (Fig. 6; KW-H (2, *n* = 36) = 25.5195; *P* < 0.001). The predominantly negative consumption rates for both algae (water uptake and autogenic effect outbalanced the mass loss from consumption) corroborated our observations during the experiments, where no active feeding occurred. Instead, *H. kergueleni* used the macroalgae exclusively as settling substrate and only consumed the fish pieces with maximal rates of about 80% of its own mass per day (Fig. 6). In fact, *H. kergueleni*’ s affinity for fish is so extreme that it does not even feed on two abundant macroalgae of PC when being exposed to starvation. This is supported by the multiple comparisons of mean ranks showing significant differences in consumption rates between macroalgae and fish (*D. menziesii* and *N. coriiceps; p* < 0.0001; *P. decipiens* and *N. coriiceps, P* = 0.0017), but no difference between the two algae species (*p* = 0.4227).

### Mandible comparisons

**Fig. 7:**
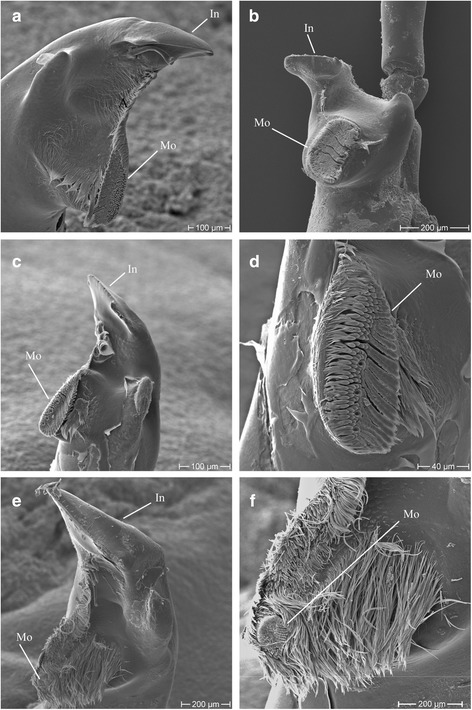
Comparisons of mandibular molars of scavenging amphipods. **a** *C. femoratus* **b** *H. kergueleni* **c** + **d** *O. rotundifrons* **e** + **f** *W. obesa*; MO: Molar; In: Incisor

The investigated species can be divided into two groups according to their mandible morphology (Fig. 7). Although all species have mandibles with smooth incisors, they differ in size with large species *W. obesa* possessing broad incisors and the small species *C. femoratus*, *H. kergueleni* and *O. rotundifrons* possessing narrower incisors. The mandibular body (corpus mandibulae) is slender in *C. femoratus*, *O. rotundifrons* and stout in *H. kergueleni*. In contrast, *W. obesa* bears a relatively larger mandibular body. Other differences between species can be seen in their mandibular molars. *C. femoratus* and *O. rotundifrons* have a similar narrow, triturating molar surface. *H. kergueleni* has an oval molar in which the triturative surface is remarkably well developed. Overall, this suggests that the mandibular of the small lysianassoid species serve a grinding function [15, 17, 36]. In contrast, the molar of *W. obesa* is a setose ‘tongue’ bearing only a vestigial triturating surface distally, indicating that this species swallows or rather gulps large food items directly into the oesophagus. A grinding mandibular molar surface crushing the food in smaller particles is hence of minor, if any, importance for *W. obesa*. The variations of mandibular molar surface of the smaller species suggest a different feeding strategy in which food particle first needs to be ground up before they can be digested. The important commonness between all investigated lysianassoid species is that all are able to cut off bites with their incisors.

## Discussion

### Functional groups among carrion-feeding lysianassoid amphipods

Our behavioural observations and analyses of feeding marks on fish carcasses suggest niche partitioning between large and small lysianassoid amphipods attending a fish carcass. While the larger species *W. obesa* is exploiting the carcass from its outside to the inside, the smaller species feed on the carcass, following an inside-outside strategy, particularly when the larger *W. obesa* is absent. Clearly, the small species lack the ability to penetrate the scale-covered skin and burrow into the carcass hence using the orifices such as anus or gills as an entryway. This behaviour is supported by the more flexible, slender and bilaterally more flattened exoskeleton of the smaller species compared to the more rigid calcified exoskeleton and round body shape of *W. obesa*. Additionally, the narrow incisors in combination with the shape of the mandibular body (corpus mandibulae) of the small species indicate that the force required for cutting through skin and scales is limited [15, 36].

Likely, burrowing into carcasses might also be a strategy to avoid predators by using carrion as a refuge [37]. *Tryphosa nana* (Krøyer 1846) (synonymised names: *Orchomene nanus*; *Orchomenella nana*) [38], a small obligate scavenger (< 6 mm) and specialised on crustacean carrion [39–42], enters crab carcasses by their limbs, body cavities, or injuries of the exoskeleton. In this way, the smaller species gain access to a food source, inaccessible for larger animals. A similar case may apply in our present study to the intestines of fish, to which the smaller species have a better access than the larger one. This means the sympatric species might avoid competitive exclusion by partitioning their common niche of scavenging. Although the niche remains the same, it is partitioned by different feeding strategies during carcass feeding. One strategy amongst others, which are discussed further below, might be the specialisation on different parts of the carcass.. This niche partitioning allows the co-existence of the scavenging amphipod species while sharing the same food source.

In the terrestrial realm functional partitioning of co-existing scavengers during carcass feeding are particularly well-known in vultures. The Old World vultures (Aegypiinae and Gypaetinae) are classified into functional types whereas big species are the tearers, medium-sized species acting as pullers and small species are considered as pickers [6, 43]. Some vulture species are specialised to feed on soft tissue and intestine starting to open carcasses by the anus and eyes while others are able to penetrate the hard skin [43–45]. In the case of scavenging arthropods, functional roles and succession patterns on carcasses are, due to the long history of forensic investigations for e.g. to estimate postmortem intervals [46–51] better understood in the terrestrial than the aquatic realm. In the marine realm the succession of deep-sea scavenging fauna on large food falls has been studied [10, 11], however, to the best of our knowledge species-specific functional roles were not reported in these studies. This might be due to the already hampered species identification of lysianassoid amphipods caused by their conservative and mainly very slight morphological diagnostic characteristics. In addition, the accuracy of identifications via images of baited cameras and their limited resolution is not sufficient. In the forensic studies of Anderson and Bell [9, 52], in which pig carcasses were sunken in the Salish Sea (British Colombia, Canada), the authors reported that ‘orchomenid’ amphipods were not able to break the skin of the carcass. Instead, the pandalid shrimp *Pandalus platyceros* Brandt, 1851, as well as the *Metacarcinus magister* (Dana, 1852) shredded the skin and subsequently ‘orchomenid’ amphipods removed the inner body tissue. Since identification of the amphipods was not a result of their study, species identities remain unclear. However, Anderson and Bell [9, 52] showed that decapod crustaceans play an important role for opening carcasses to small-sized amphipod access. With only 12 recorded species of reptant decapods in the SO that were restricted to waters warmer than 0 °C [53] and only 23 shrimp species, of which only seven are thriving in areas shallower than 500 m [54], the decapod fauna in the SO is impoverished compared to other oceans. So far only *Notocrangon antarcticus* (Pfeffer 1887) and *Chorismus antarcticus* (Pfeffer 1887) have been recorded from King George Island, of which none has been reported as a scavenger [55, 56]. Hence, so far evidence for a potential decapod species functioning as carcass “opener” is lacking in PC, and small lysianassoid amphipods either enter through body cavities or depend on *W. obesa* as a “carcass opener”. It is unknown at present if other benthic invertebrates, e.g. the giant Antarctic isopod *Glyptonotus antarcticus* s.l., may fill the void as carcass opener in the absence of reptant decapods in PC and, by extension, shallow-water benthic habitats in the Southern Ocean. Interestingly, despite the fact that we could detect a clear role allocation of small and large amphipod species during carcass feeding, our data can neither entirely reject nor confirm our hypothesis that synergistic effects between scavenging amphipods increase the speed of decomposition of fish carcasses. This might be due to our experimental conditions. Small changes in the experimental setup, such as increasing the sample size, number of amphipods, or shorten the duration of the feeding assays to reduce autogenic effects may lead to clearer statistical result.

Scavenging amphipods from the deep sea are classified into two functional groups inter alia based on their mandible morphology [16]. The first functional group is characterised as voracious, rapid feeders, processing food in separate larger bites, large and flexible guts, and the ability of surviving long starvation periods. In this group, large species, like *Eurythenes gryllus* (Lichtenstein in Mandt 1822*),* are included that have mandibles with a cutting incisor and non-triturative molars [16]. However, species of the genus *Eurythenes* do indeed have a vestigial patch of triturating molar see [57]. Members of the second functional group are less rapid in the feeding process. They have rather a continuous feeding behaviour and likely do not survive long starvation periods. This group includes smaller species as members of the *Orchomene* complex (‘orchomenids’) bearing mandibles with a triturative molar and a slender incisor [15]. Likewise, the classification of deep-sea species can be applied to shallow water scavenging amphipods. Accordingly, the small species *C. femoratus*, *H. kergueleni* and *O. rotundifrons* can be assigned to the second functional group as they all have triturative molars and slender incisors. *W. obesa* can be categorised in the first functional group due to its broad incisor, vestigial triturative molar, larger size and large stomachs [14, 58]. Also this species is known to survive starvation up to 18 months [58]. However, we cannot confirm that smaller species, particularly not *C. femoratus*, are less voracious than the larger ones. Based on our observations, both size groups of amphipods appear to have non-feeding phases in which they rest, but the smaller species seemed to swim more actively. This indicates that also differences in metabolic rates and other physiological parameters such as respiration between small and large species *W. obesa* exist [59, 60]. Our morphological results support previous studies on morphological adaptations and feeding strategies of amphipods based on their mandible morphology [17, 36, 61]. It can now be assumed that small and large scavenging species cannot solely be differentiated according to their morphological characteristics such as body size, mouthparts or gut size, but also due to their function during carrion feeding and thus carcass decomposition.

### Ecological role of scavenging amphipods in Antarctic coastal food webs

It is an undisputed fact that scavengers are of utmost importance in ecosystem functioning, particularly in food webs. The distribution and trophic transfer of resources in nutrient cycles are driving factors of evolutionary and biodiversity patterns. However, scavengers are largely underestimated (16-fold) regarding their mean percentages of links in food webs [62]. In order to identify feeding links, intensities and frequencies in food webs, information on the species-specific scavenger state and feeding rate are essential. This needs to be considered particularly in food web models. The number of trophic components [63] and individual consumption rates significantly affect the stability and dynamics of food webs. Thus, it is ultimately not sufficient to include a single indiscriminate scavenger group in food web models. Furthermore, accurate and explicit species identifications are compulsory in order to characterise species-specific food web analyses.

Our results indicate that *C. femoratus* preferably consumes fish tissue, but can also switch to omnivorous feeding, if need be, by consuming the macroalga *P. decipiens,* which was shown to be palatable to sympatric consumers [64]. This is in agreement with other studies [33]. In contrast to our results, however, Lastra et al. [33] detected higher feeding rates for *C. femoratus* on *P. decipiens* than on fish muscle. This might be due to a lesser palatability of non-indigenous fish as they used hake muscle (*Merluccius* spp.) in their feeding trials, a genus which is alien to the Southern Ocean [65]. Furthermore, artificially produced food items, i.e. extractions/pellets in feeding trials, [66, 67], are of limited use for estimating consumption rates. Feeding trials conducted under laboratory conditions are per se not an exact reflection of reality. Instead, the investigated conditions should be as close to natural conditions as possible and thus local natural food sources should be used.

### Historical versus ecological biodiversity patterns

Habitats that have been subject to fundamental environmental change often show corresponding patterns of decreasing species diversity, because recovery and colonisation processes are happening at finite speeds and require time to reach a new equilibrium [68, 69]. This observation is true across many temporal and spatial scales. Benthic diversity patterns in sponges and bryozoans on the shelf of South Georgia showed a pattern of decreasing taxonomic richness inside of the hypothesized boundary of glaciation [69]. This may be a historical signal of (surprisingly slow) recolonisation after the glacial retreat [69]. On a smaller spatial scale, a recent study about the scavenging amphipod species composition in PC [24] observed a similar decreased reduction in species diversity with the sharpest break occurring at the boundary between ice-free (outer cove) and glaciated habitat (inner cove) during the last neoglacial period [24, 25]. Although about ten lysianassoid amphipod species occur right up to the moraine separating inner and outer cove, only *C. femoratus* occurs in high abundance in the inner cove section, still dominated by glacial influence of ice and meltwater input [24]. This finding is superficially similar to what Poulin et al. [68] have termed as ecological successful colonisers after the Last Glacial Maximum. However, their main distinction was that species with pelagic larvae should be better-equipped colonising newly available ice-free areas than more sedentary brooding species and hence should become more abundant inshore than brooding species. In the case of the lysianassoid amphipods in PC, it does not seem that the difference in dispersal capability would explain why *C. femoratus* would outcompete the other lysianassoid species inshore, as all amphipods are brooders. The successful coloniserof the inner cove *C. femoratus* is semelparous whereas species that only occur in the outer cove such as *W. obesa* and *H. kergueleni* are iteroparous. Reproductive strategies are unlikely to be the single favourable trait responsible for *C. femoratus*´ colonisation success as the reproductive rate in the latter two species is higher [70–72]. Instead the selective advantage may lie in the dietary flexibility of *C. femoratus* being able to switch to lectivory when the preferred food source (dead fish) is not available. This flexibility was not observed in *W. obesa* or *H. kergueleni,* other lysianassoid candidates to colonise the inner cove from the outer cove own observation, [14, 58, 70, 73–76].

The persistence of the pattern of *C. femoratus* dominating the abundance of lysianassoid amphipods in the inner cove further supports the assumption that this pattern may not be driven by historical processes alone. It is hard to image that this pattern persisted over a few kilometres and about 2.6 to 1.6 cal kyr BP [25] given that scavenging amphipods are highly motile and attracted over long distances to the nearest food fall. The more flexible feeding ecology of *C. femoratus* would provide this species with a persisting advantage in a challenging environment and thus explain the stability of the dominance of *C. femoratus* more convincingly.

Species colonising newly ice-free habitat are destined to become a common theme in the Antarctic as ongoing climate warming, especially in the West Antarctic, will expose large areas of Antarctic shelf that have up until recently been covered by ice e.g. [77]. The collapse of the Larsen A, B and C ice shelves at the Eastern coast of the Antarctic Peninsula already initiated the colonisation of pioneer species and biodiversity shifts [78] with more shelf areas becoming exposed in the near future. Climate change-related biodiversity redistribution and range extensions open up new and large-scale opportunities for the recruitment of non-native, invasive species that have the potential to affect ecosystem functioning and ecosystem services [79].

## Conclusion

Carcass feeding strategies differ between members of the scavenging amphipod intraguild of PC revealing ‘outside-inside’ feeders (opener) and ‘inside-outside’ feeders (squeezer). Morphological adaptations favour a niche partitioning of large-sized and small-sized sympatric species of lysianassoid scavengers of PC. It appears, however, that the existence of functional groups does not influence the biomass turnover rate. The feeding strategy of the most abundant lysianassoid scavenger in PC (*C. femoratus*) demonstrates its opportunistic behaviour with relatively high feeding rates on both fish and algae (*P. decipiens*) while fish (*N. rossii*) is preferred if available. In contrast, *H. kergueleni* fed on fish (*N. coriiceps*) exclusively and showed no consumption of macroalgae (*P. decipiens, D. menziesii*). These findings suggest that a high flexibility in utilizing different food sources might confer an advantage in (re)colonisation of new habitats.

Furthermore, these findings indicate that while historical factors may determine the overall reduction of taxonomic diversity in recently ice-free newly available habitats, ecological factors such as the omnivore feeding strategy of *C. femoratus* may determine which of the regionally available candidates for colonisation is most successful. This study is giving us a better understanding on structures of scavenger (intra)guilds, their function, and importance and how speciation processes could shape the scavenger guild of Antarctic coastal ecosystems. Questions remain regarding the responses, resilience and successive replacement of *C. femoratus,* the amphipod and scavenger guild as a whole when ongoing climate-driven environmental changes may lead to community shifts [21]. This may have cascading effects in the food web and thus impacts on the entire ecosystem [79].

## Additional file

Additional file 1: Synopsis of behavioural observations and feeding marks of lysianassoid amphipods during carcass feeding. (DOCX 45 kb)

## Abbreviation

PC: Potter Cove
