# Supplementary material for: Different feeding strategies in Antarctic scavenging amphipods and their implications for colonisation success in times of retreating glaciers
Source: Front Zool. 2017 Dec 27;14:59. doi: 10.1186/s12983-017-0248-3 (PMC5745984; doi:10.1186/s12983-017-0248-3)
Supplement: Additional file 1: — Synopsis of behavioural observations and feeding marks of lysianassoid amphipods during carcass feeding. (DOCX 45 kb) [file 12983_2017_248_MOESM1_ESM.docx]

**Synopsis of behavioural observations during carcass feeding experiment. Treatment 1: large-sized scavenging lysianassid species *Waldeckia obesa***

| **Duration** | **Replicate 1** | **Replicate 2** | **Replicate 3** | **Replicae 4** |
| --- | --- | --- | --- | --- |
| **0 h** | all amphipods sitting on mesh, no movements | all but two specimens sitting on mesh, no movements | all specimens sitting on mesh, no movements | all but three specimens sitting on mesh, two swimming |
| **12 h** | specimens distributed ± evenly on carcass from head to caudal fin, mainly along the dorsal fin; several big feeding traces clearly visible (middle dorsal to posterior half of body) and caudal peduncle; 7 specimens not on carcass | 12 specimens sitting on mesh, others at mouth, dorsal fin and right pectoral and pelvic fin. Feeding traces visible at right head part, along dorsal fin area and caudal peduncle | 13 specimens sitting on mesh; others at head (mouth, burrowing in left eye), pectoral fin, dorsal fin and caudal fin. Feeding traces visible at dorsal fin and caudal peduncle | 7 specimens sitting on mesh, others on carcass (carcass fallen over to right body side), specimens feeding along dorsal fin, head (dorsal and lateral part). Big fleshy feeding trace at ventral side of body, along ventral fin, operculum, dorsal fin and above left pectoral fin |
| **24 h** | n.a. | 9 specimens, sitting on mesh, swimming; others on head, dorsal fin, right lateral body and sitting at pectoral and pelvic fin; feeding traces at dorsal head, between eyes and nose; right lateral body (only skin eaten off), caudal peduncle, dorsal fin (both fleshy) | 16 specimens sitting on mesh, others at head: left eye, right operculum, mouth, dorsal front one specimen on caudal fin. Skin is falling off at several parts including rays; deep feeding traces along dorsal fin, pectoral fin, caudal peduncle | 5 specimens sitting on mesh, others distributed on carcass, head, dorsal fin, ventral fin, caudal peduncle and caudal fin, similar to 12 h earlier. Intensive feeding trace on caudal peduncle; several parts of skin have fallen off. |
| **36 h** | 16 specimens sitting on mesh, only some (four) at head; majority at caudal peduncle, deep feeding trace at anterior dorsal fin and caudal peduncle | Two specimens sitting on mesh; one half amphiopds agglomerated at mouth, others along dorsal fin and caudal peduncle; deep feeding trace on right lateral body | 15 specimens sitting on mesh; the rest but four (one caudal fin, three at dorsal fin) at head: lips, nose, ventrally to left eye, right operculum (burrowing); deep feeding trace ventrally to left eye; removed skin from pectoral fins. Generally, same arrangement of amphipods as 12 h earlier indicating that no amphipod has moved from its position | Eight specimens sitting on mesh. Generally, same arrangement of amphipods as 12 h earlier indicating that no amphipod has moved from its position. Same holds true for feeding traces |
| **48 h** | 14 specimens sitting on mesh; rest ± evenly distributed on carcass; deep feeding traces on caudal peduncle | One specimen sitting on mesh; similar arrangement of amphipods as 12 h earlier, only some more at mouth area; more feeding traces visible at mouth and nose; cranial bone visible | 12 specimen sitting on mesh, all but three (one caudal fin, one anterior dorsal fin, one posterior dorsal fin) at head, left operculum opening, tips of branchiostegal rays skinless, jaws, lips, rays of dorsal pectoral and caudal fins become more skinless | Five specimens sitting on mesh, others ± evenly distributed on carcass as 12 h earlier; posteroventral feeding trace increased in size |
| **60 h** | 11 specimens sitting on mesh; rest rest ± evenly distributed on carcass, no aggregation; rays of dorsal and caudal fin skinless | One specimen sitting on mesh; others aggregating at mouth/nose area; others scattered along dorsal fins | Eight specimens sitting on mesh; aggregation on left buccal area; few feeding beneath operculum; bone of ventral jaw on left side visible, bone of preoperculum of right side skeletonised | Eleven specimens sitting on mesh; smaller aggregation at mouth and caudal peduncle; size of ventral feeding trace increased; rays of ventral, pectoral and caudal fin skinless; bone of left buccal area skeletonised, feeding trace dorsolateral; dorsoposterior specimen burrowing |
| **72 h** | Seven specimens sitting on mesh; small aggregation on right buccal area and caudal peduncle; big fleshy feeding trace at dorsal caudal peduncle; bone of maxilla skeletonised, rays of both pectoral fins skinless; bone of buccal area on right side skeletonised | One specimen sitting on mesh; agglomeration at mouth, anterior dorsal fin and posterior dorsal fin; more feeding traces dorsally visible | One specimen sitting on mesh; small aggregation on left buccal area, others distributed along dorsal fins and jaws | Eleven specimens sitting on mesh; aggregation at head and caudal peduncle; two specimens feeding on left lateral body; ventral feeding traces increased in size |
| **84 h** | Four specimens sitting on mesh; one swimming, rest ± evenly distributed on carcass | no specimen sitting on mesh; no aggregation at mouth anymore but at caudal peduncle, others along dorsal fins; maxilla and nasal bone skeletonised | One specimen sitting on mesh; one swimming; aggregation on left buccal area and mouth, others at posterior dorsal fin, caudal peduncle and pectoral fin | Ten specimens sitting on mesh; aggregation at mouth/head and caudal peduncle; few on left lateral body; no changes in feeding traces |
| **96 h** | Five specimens sitting on mesh; aggregation at caudal peduncle; others at anterior dorsal fin, left and right buccal area of head | Two specimen sitting on mesh; one swimming; aggregation at caudal peduncle; others anterior dorsal fin and few at posterior dorsal fin, few on right side of buccal area | Four sitting on mesh; one swimming; aggregation on left buccal and mouth area; few on caudal peduncle; one at dorsal fin | Nine specimens sitting on mesh; aggregation at mouth/ head and caudal peduncle |

**Synopsis of feeding marks on fish carcass after 96 h. Treatment 1: large-sized scavenging lysianassid species *Waldeckia obesa***

|  | **Replicate 1** | **Replicate 2** | **Replicate 3** | **Replicae 4** |
| --- | --- | --- | --- | --- |
| **After 96 h** | **Left body side:** preoperculum deep feeding trace (length x width = 2.6 x 2 cm); posteriorly to mouth, eye hollowed; nasal area, smaller marks lateral, several bigger dorsal caudal peduncle  **Dorsal:** anterior dorsal fin (2 x 1.5 cm), several at posterior dorsal fin (0.5 to 1cm); sunken eyes, traces at nostrils  **Right body side:** premaxilla skeletonised; nostril, preoperculum area (buccal) 2.5 x 1.5 cm, branchiostegal rays, dorsoposterior area of operculum opening, radials of pectoral fin, rays of pectoral fin; dorsolateral several small tracwes from 0.6 to 1.2 x 0.4 cm to 0,8 cm; caudal peduncle 2.5 cm long x half way peduncle; neural spines skeletonised  **Ventral:** small caudal peduncle ventral fin | **Left side:** eye sunken but entire; nasal, premaxilla and maxilla skeletonised; teeth visible, minute trace on operculum; several small (0.5 to 0.7cm) dorsally on lateral side; posterior dorsal fin (2.6 x 1 cm); minute caudal peduncle  **Dorsal:** anterior dorsal fin (0.7 x 1 cm); head almost completely skeletonised; several minute marks along posterior dorsal fin and cuadal peduncle  **Ventral:** dentary (mandible) skeletonised  **Right body side:** maxilla and nasal skeletonised; sclera present but empty; 2 x 2 cm hole poesterolateral of eye ( skeletonised; 1,5cmx0,5cm mark on operculum; branchiostegal rays skinless; procoracoid (0.5 x 0.5cm); rays of pectoral fin skinless; caudal fin (2 x 1 cm); level of lateral line three deep but small (0.5 x 0.5cm) | **Left side:** eye sunken but entire; around eye deep marks; buccal area widely skeletonised; maxilla and mandible skeletonised; tips of branchiostegal rays skeletonised; procoracoid (0.7 x 0.7 cm); pectoral fin slightly skeletonised; caudal peduncle (1.5 x 0.8 cm; 0.6 x 0.7cm)  **Ventral**: between maxilla and branchiostegal rays (3 x 1cm; 0.5 x 0.5 cm; 0.75 x 0.5 cm); tips of branchiostegal rays skeletonised  **Right body side:** eye sunken, eyeball empty; buccal area (2 x 1.5 cm) mark skeletonised; posterior margin of operculum skeletonised; dorsal to pectoral fin (0.5x 0.5 cm); dorasl fin skeletonised  **Dorsal:** anterior dorsal fin (1.5x 1 cm); along posterior dorsal fin continous minute feeding traces | **Left side:** buccal area (1.5 x 1.5 cm) skeletonised; eye sunken but present; premaxilla and dentary partly skeletonised; posterior to eye (0.5 x 0.5cm; 1.5 x 1.5 cm) dorsally to anterior dorsal fin (0.5 x 0.5 cm; 1.2 x 1 cm ); dorsal to pectoral fin (0.5 x 0.5 cm); dorsolateral along dorsal fin (1.3 x 0.9 cm; 0.8 x 0.5 cm) skeletonised, neural spines visible and partly skeletonised; dorsal and ventral caudal peduncle (1.3 x 0.5 cm) caudal fin not skeletonised  **Dorsal:** nostril skeletonised  **Front:** premaxilla, dentary and maxilla skeletonised up to gap between nostrils skeletonised, teeth visible  **Right body side:** buccal area (0.9 x 1.9 cm, 0.8 x 1.1 cm); posterior dorsal fin (1 x 1cm; 2 x 0.5 cm); neural spines visible |

**Synopsis of behavioural observations during carcass feeding experiment. Treatment 2: small-sized (*C. femoratus*, *H. kergueleni*, *O. rotundifrons*)**

**and large-sized (*W. obesa*) amphipod species**

| **Duration** | **Replicate 1** | **Replicate 2** | **Replicate 3** | **Replicae 4** |
| --- | --- | --- | --- | --- |
| **0 h** | many small amphipods actively swim some large amphipods also; majority sitting on mesh; some small 'attacking' directly caudal fin, after some seconds large amphipods followed to sit down on carcass | many small amphipods actively swim, some large amphipods also; majority sitting on mesh, within few seconds small start to sit on caudal fin and rest of body | many small amphipods actively swim, few large amphipods also; majority sitting on mesh. Within seconds small ones attack mouth area | many small amphipods actively swim, few large amphipods also, some small float at water surface, majority of small ones swim, none on mesh, contrary to large ones, within second start sitting on entire carcass |
| **12 h** | majority sitting on mesh, others at mouth; rays of dorsal, pectoral, pelvic and caudal fin skeletonised | majority sitting on carcass, some small on bubble stone, aggregation of small and large on mesh; aggregation of small at anterior dorsal fin, another one at posterior dorsal fin; large amphipods rest ± evenly distributed on carcass; small inside left eye and nasal area; rays skeletonised on all fins | majority of large amphipods on mesh; aggregation of small inside left eye; large randomly distributed, some ventral side of head; rays skeletonised on all fins; left buccal area becoming skeletonised | majority of large amphipods sitting on mesh; big aggregation of small at right operculum and pectoral fin, some at caudal peduncle, aggregation inside right eye; aggregation of small and big on floor; nasal bone skeletonised, branchiostegal rays on the right side skeletonised; left eye falling out; anterior dorsal fin and dorsolaterally of right body side fleshy feeding traces |
| **24 h** | n.a. | Some small amphipods on bubble stone, at surface and swimming; aggregation of large and small sitting on mesh, at mouth, at posterior dorsal fin; aggregation of small at anterior dorsal fin; few large sitting on pectoral fins and caudal peduncle | 29 large amphipods sitting on mesh; few small ones; few small swimming; big aggregation of small amphipods inside left and right eyes and buccal area, others on caudal fin; large distributed on branchiostegal ray, ventral fins; feeding traces at caudal peduncle and dorsals side of left pectoral fin | aggregation of 20 large and some small amphipods on mesh; few small and five large swimming; big aggregation of small amphipods inside right eye; some underneath the skin of „neck“; feeding traces on dorsal area, lateral right body side, right operculum, caudal peduncle |
| **36 h** | few small swimming, big aggregation of small on mesh; 15 large on mesh; aggregation of small inside right eye, ventral jaw (right), underneath operculum; large distributed ± evenly: dorsal, ventral pectoral fins, mouth; branchiostegal rays on right side skeletonised, fleshy feeding traces at caudal peduncle, posterior dorsal fin, lip of maxilla | aggregation of small amphipods on mesh, 16 large amphipods on mesh; few small on bubble stone; larger aggregation on mouth area, posterior dorsal fin (mainly small amphipods) and caudal fin (large amphipods); feeding traces along dorsal fins | 22 large amphipods sitting on mesh, few small also; some small swimming; left lateral head covered with small amphipods and six large. Sclera swinging outside the eye; aggregation at caudal fin; some small underneath left operculum; some at posterioventral fin  feeding traces: caudal fin, caudal peduncle | few small amphipods swimming; 14 large on mesh; aggregation of small on mesh; big aggregation of small amphipods on anterior dorsal fin; aggregation of large and small on right buccal area; small amphipods inside eyes; some small and large isolated at ventral fin; feeding traces: two big and fleshy marks es at right dorsolateral side with small amphipods inside; left sclera hanging outside the eye |
| **48 h** | majority of small in a big aggregation on mesh; twelve large on mesh; aggregation of small inside right eye, underneath right operculum; large distributed ± evenly on carcass | some small and large swimming, 15 large on mesh; smaller aggregation of small as well; distribution on carcass similar to 12h earlier, no changes | 14 large sitting on mesh, few small ones only; few small swimming; aggregation at caudal fin, dorsal fin and left buccal eye (mainly small, some large) some large ventral side of head | some small amphipods swimming; big aggregation of small and 17 large on mesh; big aggregation of small at anterior dorsal fin, smaller agglomeration at caudal fin; some small in right eye and underneath skin dorsalposterior head; small agglomeration of large at right buccal area; some small underneath skin ventral fin |
| **60 h** | 21 large amphipods sitting on mesh, two aggregation of small as well; small amphipods inside right eye, underneath operculum and lateral bone window, underneath branchiostegal rays, head, few specimens on body, few swimming; large amphipods scatterd on carcass (head, right lateral side). Feeding traces: small underneath skin of head | Twelve large amphipods sitting on mesh, only few small;­ big aggregation of small at caudal fin; aggregation of small and big at posterior dorsal fin; small group of big at nasal bone; small inside eye; feeding traces increased at posterior dorsal fin | 14 large sitting on mesh, some small as well; some small swimming; aggregation of small at caudal fin; small indside left and right eye; on/underneath skin of buccal area; some large at mouth, branchiostegal area and left lateral side | 17 large amphipods on mesh; bigger aggregation of small also aggregation of small at dorsal fins, underneath skin posterior head; one large and many small inside left eye; further large on left buccal area; some sitting on caudal fin (three large, few small); some small inside feeding traces of right lateral side |
| **72 h** | majority of large amphipods on mesh, two aggregations of small as well; some small and large swimming; ± same distribution as 12 h earlier; small underneath operculum, branchiostegal rays area; large scattered on body, upper jaw, anterior dorsal fin | Twelve large amphipods sitting on mesh, aggregation of small as well; aggregation of small and large on posterior dorsal fin; group of large on nasal bone; small inside left eye; some scattered; small underneath skin | Eleven large amphipods sitting on mesh, few small also; one large swimming; ± same distribution as 12 h earlier; aggregation of small at caudal fin, inside left and right eye, left buccal area underneath skin; large amphipods lower jaw and inside mouth | 16 large amphipods sitting on mesh; very few small; some small swimming; big aggregation of small on dorsal fin, small agglomeration on caudal fin, inside left and right eye; underneath skin behind head; large on jaws, nasal bone |
| **84 h** | Some small swimming, aggregation of small on mesh, several large as well; large on dorsal head, nasal bone, branchiostegal ray, jaws; some large scattered on body; small either inside fish (not visible) or on mesh | Nine large amphipods sitting on mesh, few small as well; some small amphipods swimming; more distributed on dorsal parts, and caudal peduncle | Small agglomeration of large amphipods on mesh (15); few small amphipods swimming; big aggregation of small on caudal fin, inside left eye, inside mouth; maxilla bone skeletonised | 13 large amphipods sitting on mesh, few small; some small and one large swimming; aggregation on anterior dorsal fin and caudal fin; small underneath skin in pockets; some also on right pectiral fin; aggregation inside eyes; large along maxilla and nasal part of head |
| **96 h** | big aggregation of small and large amphipods sitting on mesh; few small swimming; group of large on nasal, maxilla and operculum; only few small inside eye (right); no small amphipods visible on carcass | Ten large and some small sitting on mesh; several small swimming; small and at caudal peduncle; small also in skin pockets along dorsal parts, small inside eyes, large feeding on maxilla | 15 large and few small amphipods swimming; aggregation of small at caudal fin, inside eyes and buccal area; large on dentary (lower lip) and ventral head; teeth skeletonised; some large feeding dorsalposterior head (anterior dorsal fin) | Few small amphipods swimming; ± distribution as 12 h earlier; few large on mesh almost non of the small ones |

**Synopsis of feeding marks on fish carcass after 96 h. Treatment 2: small-sized (*C. femoratus*, *H. kergueleni*, *O. rotundifrons*)**

**and large-sized (*W. obesa*) amphipod species**

|  | **Replicate 1** | **Replicate 2** | **Replicate 3** | **Replicate 4** |
| --- | --- | --- | --- | --- |
| **After 96h** | **Left side:** eye absent, part of sclera remnants in eye, premaxilla partly skeletonised  **Ventral:** feeding trace basis pectoral right fin (2 x 1 cm; 1.5 x 0.5 cm) abdominal part between pelvic fins; anus (1 x 1 cm, deep), several smaller marks along ventral fin; branchiostegal membrane perforated  **Right side:** eye absent, only part of sclera still present, nostril, dentary and premaxilla partly skeletonised; branchiostegal rays partly skeletonised; pectoral fin (0.2 x 0.2 cm);  skin removed from caudal peduncle, dorsolateral (0.3 x 0.3 cm); dorsoposterior head (1 x 0.5 cm)  **Front:** premaxilla, maxilla nostrils, snout skeletonised | **Left side:** eye absent, completely hollowed to the other side, expanded, no sclera; premaxilla, dentary skeletonised; buccal area (0.5 x 0.7 cm), posterior to eye (0.5 x 0.5 cm); anterior dorsal fin (0.7 x 0.7 cm); beneath operculum; caudal peduncle (dorsal 0.7 x 0.7 cm) (ventral 0.5 x 0.5 cm)  **Right side:** premaxilla, dentary skeletonised; eye fully hollowed like left side ,nostril skeletonised; dorsoposterior head (0.3 x 0.6 cm); lateral head (0.3 x 0.7 cm); along posterior dorsal fin (5 cm x 0.7 cm), neural spines visible; caudal peduncle (1 x 0.5 cm)  **Dorsal:** head, between eyes skeletonised;deep marks along anterior, posterior dorsal fins (4 cm, both sides) along posterior dorsal fin (6.5 cm), deep, neural spines skeletonised  **Ventral:** anus fleshy and widened (1.5 x 0.7 cm); ventryl fin (0.2 x 0.2 cm) | **Left side:** eye hollowed; premaxilla, maxilla, dentary buccal area skeletonised; branchiostegal rays skeltonised, pectoral fins skeletonised; dorsal caudal peduncle (1 x 0.3 cm); dorsal fins skeletonised  **Ventral:** 1.5 x 1.5 cm, 1.6 x 0.8 cm, 0.2 x 0.3 cm; 0.3 x 0.2 cm branchisostegal membrane; anal area (0.6 x 2cm); along ventral fin, posteriorly (0.7 x 0.5 cm), deep  **Right side:** like left side: eye hollowed; buccal area skeletonised; premaxilla, maxilla dentary skeletonised, operculum; branchiostegal rays skeletonised; posterior to operculum, on pectoral fin (0.4 x 0.4 cm); two mark of equal sitze dorsal pectoral fin (1.7 x 0.6 cm); posterior dorsal fin below (1 x 0.3 cm); further posterior (0.4 x 0.2 cm); caudal peduncle (0.7 x 0.5 cm)  **Dorsal:** between eyes skeletonised  **Remarks:** There is a passage from one eye to the other, and one from eye to infraorbitales to buccal area: sclera still present | **Left side:** eye hollowed; premaxilla, maxilla, dentary, nostril skeletonised; ventral side of eye (0.7 x 0.5cm); pectoral fin (1 x 1.5 cm; 0.5 x 0.5 cm); posterior side of pectoral fin (1 x 2.5 cm, deep, spines visible) 0.7x 1cm, 1x1.6cm (spines visible); 0.6 x 0.6 cm, 0.7 x 0.6 cm; dorsal fin (1.6 x 0.8 cm, neural spines visible); dorsolateral (0.6 x 0.7cm, 0.8 x 0.8 cm); caudal peduncle (0.5 x 0.5 cm)  **Ventral:** anal area: widened, (1.5 x 1 cm); ventral fin (0.5 x 0.7 cm, 1.6x1cm, 0.7 x 0.5 cm, 0.6 x 0.8 cm, 0.4 x 0.5 cm)  **Right side:** eye hollowed with passage to other side; passage to buccal area premaxilla, maxilla, dentary, skeletonised (see remarks); 0.6x1cm dorsoposterior head (1.2 x 1.2 cm); above pectoral fin spines visible, deep (1 x 1.5 cm); ventrolateral (0.9 x 1.1 cm, 0.5 x 0.9 cm, 0.5 x 0.5 cm); dorsolateral (0.3 x 0.3 cm, 0.5 x 0.5 cm; 0.7 x 0.7 cm, 0.2 x 0.3 cm; caudal peduncle (0.5 x 0.5 cm)  **Dorsal:** posterior head: passage to gills, hollowed; otherwise feeding traces described above, all deep and fleshy  **Front:** skeletonised from dentary upwards to between eyes area  **Remarks:** There is a passage from one eye to the other, and one from eye to infraorbitales to buccal area: |

**Synopsis of behavioural observations during carcass feeding experiment. Treatment 3: small-sized (*C. femoratus*, *H. kergueleni*, *O. rotundifrons*)**

**amphipod species**

| **Duration** | **Replicate 1** | **Replicate 2** | **Replicate 3** | **Replicate 4** |
| --- | --- | --- | --- | --- |
| **0 h** | some sitting on mesh other swimming and directly agglomerate at caudal fin and start to sit on entire body | some sitting on mesh other swimming and directly agglomerate at caudal fin and start to sit on entire body | almost every specimen swimming, some still sitting; starting to sit on entire body | almost every specimen swimming, some still sitting; starting to sit on entire body |
| **12 h** | two small aggregation on mesh; one big agglo at caudal tail, a small one at mouth; few swimming | one small aggregation on mesh, small one at caudal fin many others along dorsal fin line, pectoral fin and inside left eye; rays at caudal and pectoral fins visible; some swimming | aggregation on mesh; aggregation at caudal fin; some swimming; rays at all fins visible except pelvic fin; underneath operculum | no aggregation on mesh but still some sitting; few swimming; big aggregation on head lateral, pectoral fin and underneath operculum |
| **24 h** |  | some swimming, few on mesh; others widely distributed on entire body | some swimming; few on mesh; many underneath operculum and anus forming a pocket; some on the body, tips of rays becoming visible | some more swimming, only few sitting on mesh; big aggregation at right operculum, pectoral fin, entering operculum, a group at anterior dorsal fin; right eye is starting to get off; skin is fallen off patchy |
| **36 h** | few swimming; almost none on mesh, others agglomerated in corner of aq, some underneath operculum | few swimming; small aggregation on mesh, bigger aggregation at head (right eye, nasal area) and caudal fin; skin fallen off patchy | few swimming; two small aggregation on mesh; others underneath right operculum and anus, very few at caudal fin, not many amphis visible | very few swimming; small aggregation at corner of aq; very few on mesh; aggregated underneath right operculum, some dorsal fin; more skin fallen off; eye still in eye but half way out |
| **48 h** | few swimming; none on mesh; very big aggregation at caudal fin; underneath left operculum, inside mouth | very few swimming; two small aggregations on mesh; big aggregation inside right eye, some inside left eye and small aggregation at mouth/inside; some patchy distributed on body | some swimming, very small aggregated on mesh; some inside mouth; underneath operculum anus opening; majority not visible | few swiming; few on mesh; some on pectoral fins and dorsal fin; many underneath operculum |
| **60 h** | no swimmers; about 15 on mesh; very big aggregation at caudal fin, others underneath operculumn, not many visible | few swimming; small aggregation on mesh; small aggregation at right eye, mouth and few left eye; others patchy distributed; majority not visible | few swimming, small aggregationon mesh; many underneath operculum; pocket/ anus opening very big with amphipods inside | none swimming; few on mesh; big aggregation underneath operculum (right side) and pectoral fin; some dorsal fin; eye still auf half past 7 |
| **72 h** | few swimming; only about 15 on mesh; big aggregation at caudal fin; and underneath operculum; majority not visible | some swimming; aggrgation at left and right eye; some patchy distributed on entire body and fins | some swimming; no aggregation on mesh, but underneath operculumn and anus opening; majority not visible | some swimming; no aggregation on mesh, but underneath operculum; not so many as 12h before opening behind pectoral fin; underneath also amphipods |
| **84 h** | few swimming, almost none on mesh; big aggregation on caudal fin; underneath operculum | few swimming; small aggregation right eye; big one on mesh | few swiming; smaller aggregation on mesh; underneath operculum and anus; majority not visible | none swimming; some on mesh; fish turned on left body side; big aggregation at right pectoral fin and underneath operculum; some ventral visible; fish only small patches with coloured skin |
| **96 h** | none swimming; 3 on mesh; big aggregation on caudal fin; amphipods underneath operculum visible; majority not visible | some swimming, som eon mesh; Eye sclera swimming around; aggregation at caudal fin left an right eye | some swimming; some on mesh; underneath operculum; big asshole; majority not visible | some swimming; few on mesh; big aggregation at right pectoral fin and underneath operculum, others patchy distributed |

**Synopsis of feeding marks on fish carcass after 96 h. Treatment 2: small-sized (*C. femoratus*, *H. kergueleni*, *O. rotundifrons*)**

**and large-sized (*W. obesa*) amphipod species**

|  | **Replicate 1** | **Replicate 2** | **Replicate 3** | **Replicae 4** |
| --- | --- | --- | --- | --- |
| **After 96 h** | **left side:** 0.2x0.4 mark branchiostegal membrane, eye present; caudal peduncle: 0.6x0.5cm, 0.7x0.7; posterior dorsal fin partly skletnsd  **right side:** eye normal, tiny marks at cuadal pdcl:4 marks á 0.2x0.1 cm  **ventral:** anal area widened, expanded 0.7x0.9cm | **left side:** sunken, eyball empty; beneath pectoral fin entrance/opening 2cm long (at least)  **right side:** eye absent, hollowed, nostril expanded; abdomen sunken (intestines eaten (?)  **dorsal:** posterior dorsal fin 2x 0.2x0.3cm marks  **ventral:** anal area slightly widened (?) | **left side:** eye present,sightly sunken  **dorsal:** nothing  **right side**: eye present but sunken; operculum entrance dorsally widened; caudal pdcl tiny tiny marks not measurable; dorsal fin posterior part slightly skltnsd;  **ventral:** anal area: immensly widened, deepened, hollowed 1x1.5cm abdominal cavity visible | **left side:** eye present, slightly sunken, skin slightly removed but no marks visible; abdominal area sunken  **dorsal:**  0.3x0.3cm mark anterior dorsal fin; 0.5x0.2cm posterior dorsal fin  **ventral:** anal area: 1.8cmx 0.7cm opening, (riesig)  **right side:** eye present but slghtl sunken, beneath pectoral fin 1.5x0.8cm opening; skin partly removed but no further feeding traces |
